# Extracts and Gleanings

**Published:** 1875-05

**Authors:** 


### Extracts and Gleanings.

*Treatment of Fractures.*—Dr. Schwab, in *Memorabilien*, (*Cincinnati Med. Journal*,) advocates the use of albumen as preferable to "plaster of Paris" bandage in the treatment of fractures.

"In addition to the whites of six to eight eggs, there will be needed an old linen sheet from which a bandage of scultetus can be cut, a piece of pasteboard, which is always at hand in the cover of an old book, and a roller bandage from three to four yards in length.

The bandage of scultetus and pasteboard are first saturated with the albumen and the bandage carefully applied, allowing the edges to slightly overlap. This bandage should reach to the joints above and below the fracture. The pasteboard is then smoothly adjusted to the part and secured with the roller. The limb is kept in proper position by means of small bran-bags or cushions of straw.

I have used this method exclusively for twelve years, in the treatment of all fractures of the extremities, with complete success. *No shortening* or other deformity ever followed.

In fractures complicated by superficial or deep wounds, an opening is cut through the pasteboaard and bandage, to permit free access to the wound.

In cases where swelling had taken place in the injured limb, I have applied this bandage, and frequently found the swelling to completely disappear on the second or third day. The bandage and splint are then taken off and reapplied.

The following points, as demonstrating the superiority of this over any other method of treatment, are presented for consideration:

1. The ease and rapidity with which the articles needed can be obtained.

2. The ease of application, and rapid drying of the bandage.

3. The early abatement of the pain.

4. The more rapid recovery, and consequently the earlier use of the fractured limb."

We make no issue with Dr. Schwab, as to the virtues of the above plan, but see no advantages in it over the *starch bandage* once so common in this country, and which we think has very improperly gone into disuse with a majority of our surgeons. *Starch,* or *paste* made with flour, will accomplish all that is claimed for the albumen in the above plan. For twenty years we have used the starch bandage in numerous cases of fracture *to the exclusion of all other plans, and without a single failure.* Opposition to this bandage first grew out of the fact that when applied as an "immovable apparatus," it sometimes occasioned gangrene by arresting the circulation. This we have always guarded against by splitting the bandage as soon as it became dry, and in some instances where the swelling was rapid and painful before it became entirely dry.—When thus split it may be sprung open and the limb examined, and the fracture readjusted if necessary. Or if the fracture be compound, a portion of the bandage may be cut away, thus leaving the wound exposed for dressing, &c.

Immediately after the bandage is put on, a temporary splint of wood should be applied to remain until the starch is dry, when the dressing will be found sufficiently firm, constituting a perfect box, which, moulding itself to the shape and irregularities of the limb, renders counter-extrusion appliances unneccessary. The box may be closed and made fast by strips tied around it, or becoming too loose by shrinking of the limb, may be made smaller by paring off its edges. In short, the *starch* or *paste* bandage possesses every possible advantage of facility in the procurement of the material, simplicity of application, neatness and comfort; and we believe when properly applied will give results equal to, if not better than, any or all other devices which have been tried or recommended in this country, or in Europe, in the treatment of fracture.

Our plan of applying the bandage is as follows: Having prepared strips of thin pasteboard two or more inches in width, according to size of the limb, and a quantity of starch somewhat thicker than that used for starching clothes, (or if no starch be at hand substitute flour paste about the consistence used in putting on wall paper,) let the limb be carefully supported by one or more assistants, and a roller bandage applied from the lower end of the limb to the joint above the fracture firmly and smoothly, but not tight. Let this be smeared with starch, and a layer of patent lint, or tailor's wadding be then smoothly applied, and smeared with starch. Then dipping the strips of pasteboard in hot water to soften them, adjust them upon the wadding, and smearing with starch bring down the roller over them with tolerable firmness, and secure by pins at the lower end. Then apply the temporary wood splint. If no patent lint or wadding be at hand, it is better to apply a second roller before putting on the pasteboard. In some instances an additional roller may be needed to strengthen the apparatus, but usually the two as above described will be found sufficiently strong. R. C. W.

*New Method of Treating Typhoid Fever.*—Dr. Henry Blanc, surgeon-major to H. M., Indian army, gives us in the *Lancet*, a new mode for treating typhoid fever. After a review of Dr. Brand's method which is based on the theory of fermentation, and acts by keeping the temperature below the fermentative point, in the following manner: At the end of the first or beginning of the second week, Dr. B. places the patient up to his neck in a bath at 68° F., he then gives him a douche of water, at from 43° to 47° F., for two minutes. An assistant then rubs, and shampoos his limbs, under water, for three or four minutes. The patient is then allowed to remain quiet for fifteen minutes in the bath, where he must stay for that length of time, no matter how much he may chatter his teeth, and show signs of intense cold. He is then taken out, and will be found able to stand with some support; his shirt is put on without wiping him, and he is given a cup of tepid broth, and a mouthful of good wine. He is now put on a hard bed, his feet wrapped in a blanket, and covered lightly. This procedure is repeated every three hours day and night till the temperature in the rectum falls to 101° F. His diet is given to him tepid, and consists of milk, tea, beef and mutton broth; a mouthful of iced water is given every half hour and cold enemas administered in some cases. Dr. Brand applies his method to all cases, in all stages of the disease; he only admits one exception, perforation of the intestine; this demands perfect rest.

The record of cures he has made, together with that of his pupils, is unprecedented. "Up to 1868, Dr. Brand treated 170 patients by the cold bath, and obtained 170 cures." Dr. Glenard of Lyons, one of his pupils, has a record of 52 cases, and 52 cures. He says, speaking of this treatment, "you will not find among the five or six thousand cases of typhoid fever treated by this method, one single unsuccess among those who have been submitted to it from the beginning of the disease."

After giving the above treatment Dr. Blanc claims that though, the results are excellent, the treatment does not cut short the disease; and that this can be done by combining the exhibition of antiseptics, with the cold water treatment. He advises calomel in the inception of the disease, and iodine, quinine, sulphuric acid, or any other powerful antiseptic during its continuance. Dr. Blanc claims that his treatment will do good service in all malarial affections.

Such is a brief summary of his article. The treatment is only new in the manner in which it is applied, as cold has long been considered one of our most powerful weapons against this terrible malady, but the results are far more favorable than any yet given. It is to be regretted that Dr. Blanc did not give us a little insight into the after life of his patients, as often the sequels of typhoid are even worse than the disease. H. B. L.

*Dr. Paul F. Munde's Treatment of Abortion.*—Hoening recommends to express the ovum, either entire or in part, if the fœtus be already removed, by means of bi-manual compression, two fingers of one hand being introduced into the vagina and passed as far up as possible into the fornix vaginæ, and the other grasping the uterus through the abdominal parietes, thus firmly compressing the organ between the fingers of both hands and slowly and surely expelling its contents. If the uterus is anteverted or anteflexed, as is usual during the earlier months of pregnancy, the two fingers should be passed into the anterior cul-de-sac, or the corpus uteri may be firmly pressed against the symphysis pubis by the external hand alone (the bladder having been emptied); if the uterus is retroflexed, the two internal fingers go behind the cervix. The relaxation of the abdominal parietetes in multiparæ (in whom, as has been statistically shown, most abortions occur), usually renders the seizure of the uterus by the external hand an easy matter. The facility and rapidity with which the expression of the ovum is accomplished, is according to Hoening, surprising and gratifying, as also the abscence of subsequent hemorrhage or puerperal trouble. The pressure in the uterus need not and never should be sufficient do harm.

Before reading this paper, I hapened to meet with a case of miscarriage, in which I succeeded in expressing the retained placenta by bi-manual compression. Since then during the past summer, I employed the manipulation in two cases, both abortions in the fourth month, in consequence of retroversion, with the most complete success. Passing two fingers into the posterior cul-desac, and grasping the uterus firmly with the other hand from without, I immediately felt the uterine contents slip out towards the cervix, and pressed them entirely into the vaginia by passing the two fingers behind the cervix forwards, towards the external os. Both ova were expelled into the vaginia entire, the membranes of one of them rupturing as it left the os. Both women made easy and rapid recoveries.

Of course it is essential that the cervix be sufficiently dilated before the measure is attempted; in my two last cases I accomplished the dilation, and at the same time controlled the hemorrhage by the introduction of the colpeurynter for several hours. The uterine contractions attending the dilatation of the os will generally loosen the adhesions of the ovum to the uterus sufficiently to allow of its easy expulsion.

The advantage of this *vis a tergo* over the introduction of the speculum, the forceps and the currette, to say nothing of the old method of passing the finger into the uterus, are obvious. I trust this communication may contribute to the general adoption of this most excellent practice.

*Tr. Mur. Ferri in Nasal Polypi.*—Dr. Maxwell calls attention, in the *Medical Times*, to the treatment of nasal polypi, by injections of tr. mur. ferri. This treatment was first brought into notice by Dr. J. H. Reeder, of Illinois. Dr. M. claims that in from one to five days, he causes the complete disappearance of the ordinary gelatinoid polypi; by injecting ʒss. of tr. mur. ferri, diluted with an equal quantity of water, into each nostril, and repeating the operation daily till sloughing occurs. As this is a very simple treatment, and an do no harm, it is worth a trial. H. B. L.

*The Hygiene of the Eyes.*—In the *Journal de Medicine* (translated in the *Medical Press and Circular*), M. Grand lays down some hygienic rules for the eye:

For the worker the light should come as much as possible from the left side, that is to say, from the side towards which one turns in working.

Daylight is the best; but one should avoid direct sunlight; that of reflecting mirrors should also be avoided. The aspect should be northern, and the sight should come a little from above. Light coming from the right, too high or low, all these defective conditions, cause school children, particularly, to take all sorts of awkward positions.

White walls should be avoided; highly varnished tables, and in workshops, shining articles like silk, should be protected from the sun's rays.

Artificial light is always bad, on account of the heat and the exhalation of carbonic acid. The best is that of lamps fed with vegetable oil and furnished with a glass shade. Gas is bad because of its heat, brilliancy, and mobility; the light of mineral oils is too hot; that of candles insufficient and flickering. An oil lamp should be covered with an opaque moderateur; the eye of the workman should avoid the light coming to him directly or diffused through the room. The moderateur should be white, green, or gray

Working after meals is injurious. Inclination of the head should be avoided. One should write on an inclined plane; and in schools it would be good to supply a movable black-board for the children. —*Med. and Surg. Reporter.*

*Singular Case of Malpractice.*—Mr. Peacock, a surgeon of London, has just been convicted of manslaughter under very singular circumstances. He delivered a woman with the forceps, and then cut off with a pair of scizzors, over fifteen feet of intestine which had escaped through a rent in the vagina. Dr. Barnes, in his testimony as an expert on the case, says that there is a case on record of a similar protrusion of the intestine, and while condemning the cutting as a rash act, he asserted that it would lengthen life by preventing shock. As this case is unique, it is worthy of a place in the RECORD.—*London Lancet—The Clinic.* H. B. L.

*Case of Abstention from Food.*—The subjoined case, reminding us of the "Welsh fasting girl," and Louise Lateau, is communicated to us by Dr. D. R. Silver, of Sidney, Ohio, who vouches for the truth of the report from his own observation. The extract is from a local paper:

"Sarah Brown, daughter of Andrew Brown, of Turtle Creek township, aged about twenty-three years, has been for five years afflicted with an obscure form of nervous disease. For the larger portion of this time she has been confined to her bed. For two years she has been totally blind, and for the same time has been wholly devoid of the power of speech. She lies at the present time helpless as an infant. Her hands are tightly closed, so that if she had muscular power, she could not help herself. At times she has severe pain, cramp and muscular contractions in various parts of her body. The strangest part of this case, however, remains to be told. At various times during her sickness her appetite has been exceedingly capricious, sometimes for three weeks taking nothing but a little milk. On the first of January of this year she refused to take any food, either solid or liquid, and absolutely continued her fast until the first of March, fifty-nine days in all, and for over fifty days her bowels were in a state of complete constipation. During this time her friends made attempts to induce her to take food or medicine, but without success. Even water having bread soaked in it, and carefully strained, was promptly rejected. Her only aliment was pure water, of which she drank freely. On the sixtieth day she was induced to swallow a little cider, and since that time she has been taking a little solid food. Incredible as this story may appear, this much is certain, that the girl cannot be a malingerer, from the nature of her case."—*Med. and Surg. Rep.*

I knew a young lady in Charleston, S. C., who, for over a month at a time, was never seen to take food of any kind. Her family tried, leaving trays of food about the room, and leaving her alone; but everything was left apparently untouched. After some days, they accidentally discovered that she would remove the top crust of bread or biscuit, and after eating out all the crumb, would replace the crust with the utmost care. May not Dr. Silver's case be a similar one? W. B. L.

*Pills of Aloes and Ipecac.*—℞. Aloes, gr. i.; ipecacuanhæ, gr. ¼; ext. gentianæ, gr. iss. M. Ft. pil. No. 1. Dose one to two pills.

*Action of Mercury upon the Blood.*—Dr. Wilbouchewitch, of Moscow (*Archiv. de Phisiologie, Bost. Med. Jour., Dec.* 1874), has made a practical application of the ingenious apparatus devised by Mahassez for counting the blood corpuscles. This apparatus briefly consists of a fine capillary glass tube of definite capacity so small as to bear a microscopic examination with an object glass capable of defining the blood corpuscles. The blood is diluted with artificial serum, placed in the tube, the corpuscles counted. By a simple calculation it is easy to ascertain the number of corpuscles in a given amount of blood.

He found that in the healthy subject the number of blood discs averaged 4,600,000 to the cubic millimetre. In different individuals it varied from four to six millions but in the same subject the number remained pretty constant.

In syphilitic subjects examined before the beginning of specific treatment, the author found a progressive decrease of blood corpuscles amounting generally to more than 200,000 daily. As soon as mercurial treatment was instituted (of one and two-thirds grains of proto-iodide was begun) the blood corpuscles began to increase in number at the daily rate of about 80,000 during the first week, and then at the rate of 120,000 daily. This increase continued during two or three weeks only. Then the number began again to diminish, so that in a fortnight the number of discs was about the same as at the beginning of treatment. If now the treatment was suspended, the destruction of the corpuscles ceased, and in about a week the blood began to grow richer at a daily rate of about 90,000. As a general rule the white corpuscles underwent variations inverse to those of the red corpuscles.

These experiments teach that the administration in syphilis should be intermittent in order that the drug shall not itself act as a destructive agent upon the blood corpuscles.—*Detroit Review of Medicine.*

*In the Cough of Chronic Phthisis.*—℞. Morphiæ acetat., gr. ii.; liquor. atropia (B. P.) *m*vi. gr. $\frac{1}{20}$ atropia; acid. hydrocyanic, dil· *m* xxv.; syr. pruni virgin. ad f℥iss. M. A measured drachm is to be taken, unmixed with water, on going to bed, and once again duing the night if necessary.

*Esmarch's Operation.*—I have already seen many of the surgical celebrities here, and have generally been better received by them than I expected an American stranger would be. Last night I attended, by invitation, a meeting of the Clinical Society, at which were present many of the leading London surgeons, and before whom Professor Esmarch, of Reil, read a short address upon his bloodless method of operating. This method was just being introduced in Califorinia when I left. A paper on this subject was read before the State Medical Society. The pith of Esmarch's paper was, that under the old plan he lost about one in three of amputations of the thigh; while under the bloodless plan he has had but one death in thirteen such operations. He has used it in nearly three hundred cases, and in no instance has any untoward event resulted from it. The plan, in short, as he described it, is this: Apply to the limb, (it can only be used on the extremities) an india rubber roller, so tight as to force the blood from the limb towards the body. Next tie a strong tubular chord of the same material at the point which the roller reaches. This done, remove the roller and the limb is left white and bloodless. Esmarch stated that in one operation he kept the limb in this state for over two hours.—The absence of blood enables you to see exactly what you are doing. The plan is being generally adopted here.

Esmarch is received with considerable *eclat* in London, so much so, that, as I am told, he is making a triumphal march among the English surgeons. He does not appear spoiled by the laurels that are showered upon him, nor is he so old, that like Cæsar, he needs to wear his chaplet to conceal calvities.

I never worked harder than I am doing here, hearing lectures and witnessing operations. Altogether I am having a good time of it. It is a delightful treat to stand back and see the hands of men known to fame do things which you have done, and to go away with a new idea for future use; though, self-praise aside, I have learned that away off yonder in our home on the sunset side of the new world, there are hands that can do things even better than I have seen them done in some instances in this metropolis, the head and the center of Anglo-Saxon culture.—*Letter from Prof. Lane to Pennsylvania Med. & Surg. Journal.*

*In Prostatic Gleet.*—℞. Copaiba *m*ii.; ess. cinnamomi, *m*xx.; mucilaginis, *m*xv.; aquæ, f℥i. M. Sig. Four times daily.

*Cincho-quinine.*—Our clinical experience in the use of the cincho-quinine having led us to think favorably of it as an antiperiodic, we confess to having experienced no little surprise when Mr. Ebert, of Chicago, stated before the Pharmaceutical Association, at its meeting in this city in August last, that the compound contained neither quinine, quinodine, or cinchonidine, and was therefore worthless. We were unwilling to allow that we had been deceived in the estimate we had formed of the powers of the drug, nor could we believe that either Mr. Nichols or Messrs. Billings & Clapp would originate a fraud. It now appears that Mr. Elbert fell in some way into an error, as a printed circular recently received contains the certificates of a number of leading chemists in different cities of the Union, in all of which it is positively affirmed that the cincho-quinine contains the several alkaloids as stated by its manufacturers. We are glad to have our good opinion of the drug sustained by such competent authority.

The above remarks, from *The American Practitioner,* express fully our experience and our views in regard to the cincho-quinine. We neither doubt the genuineness of the article nor the integrity of Messrs. Billings & Clapp, the manufacturers. R. C. W.

*Cholroform in Congestive Chills.*—Dr. R. K. Hinton writes to the *Philadelphia Medical and Surgical Reporter,* as follows :

I have been in the habit of using chlorofom in the cold stage of congestive chills, and also ordinary intermittents, for several years past, and have never found anything that would bring on reaction more speedily. The following case which I transcribe from my case-book, will show its effects :

On the fifth of July, 1874, I was called in haste to see Patrick O'Connor. The messenger informed me that he was laboring under his third sinking chill. On my arrival, I found my patient pulseless at the wrist; feet and legs cold; constant nausea and vomiting ; had had two attacks of syncope; in short, I supposed my patient would never react. Sinapisms were applied to the stomach and epigastric region, and the extremities rubbed with liquid ammonia. Hot baths could not be used, owing to syncope coming on immediately on attempting to raise him up. I immediately put him on six-drop doses of chloroform in sweetened water, to be repeated every fifteen minutes. After taking the second dose, he was much better; pulse returning at the wrist; nausea and vomiting entirely stopped. In one hour from this time, reaction was entirely established; the fever soon passed off; and after the free use of quinine, he had no return of the chills.

*Remarks.*—As I before said, I have been in the habit of using this remedy for several years past. The first was a negro boy, who was laboring under his second chill. He had obstinate vomiting; the whole array of remedies was brought to bear in this case, but to no purpose. Chloroform was substituted, and in a comparatively short time reaction was fully established.

It appears to fulfill the following indications: 1. It allays nausea and vomiting. 2. It allays the pain in the stomach. 3. It equalizes the circulation. 4. Reaction is never excessive after its use.—I have used chloroform in the hot stages of intermittent, and with the happiest results. It appears to allay nervous excitement, and by so doing, quiets the heart's action. I have also used it in the treatment of pueumonia, in from five to ten-drop doses every two hours. It allays the cough and all nervous excitement, and appears to cool down the fever.—*Med. Examiner.*

*New Principles in Surgery.*—Dr. T. Bryant, suigeon to Guy's Hospital, reports in the *Lancet* for April, some very remarkable cases of diseased bones and joints, treated by a new method; which he calls the "least sacrifice of parts." Dr. B. contends that we should never take more tissue than is already diseased, and on no account go above a joint simply to make a good flap. He takes out only as much bone as is incurably diseased, and then leaves the case to nature. In his report, he gives thirty-five cases, treated by his method, viz.: 7 of diseased bone; 28 of diseased joints; all ending in recovery, more or less perfect. In this series, embracing diseases of all the joints, except those of the spine and head, he only performed 3 amputations, and only 10 cases of anchylosis occurred, making a total of 22 perfect recoveries. In his treatment of joints, he makes free incisions, removes all necrosed bone, and gives tonics and stimulants.

The wonderful success of this method, so opposed to the general idea of the danger of opening any joint, demands the attention of the profession.

H. B. L.

*Bromide of Potassium in Malarial Fever.*—Henry J. Hilliard, M. D. of Scottsville, Harrison county, Texas, says : "I wish to call the attention of the profession to the beneficial effects of the bromide of potassium in malarial fever. I do not think that it is an antidote to the malarial poison, as are cinchona and its salts, but that it is a great auxiliary. For the past two years I have very successfully used it, both in the intermittent and remittent forms. In the majority of cases of malarial fever the nervous phenomena are quite prominent, more especially in children, whose nervous system is so susceptible of derangement. The bromide of potassium, conjoined with the local application of cold water or ice to the head, act very beneficially in such cases by relieving the delirium and restlessness. I generally give adults, during the stage of exacerbation, the following : ℞. Potassii bromid. dr. j.; spiritus nitri dulc. gtt. xv.; aquæ puræ, f oz. j ; repeat every two hours, till the period of defervescence, when quinia should be freely given ; at the same time continue the bromide at long intervals during the remission. A short time after the first dose is given, the patient generally becomes quiet, perspiration commences, and the mouth becomes moist. Cinchonism seems to be much more easily produced, absorption taking place more readily. It has greater effect over infantile convulsions during malarial fever, than anything I have ever used. Should the case be urgent, it is well to combine a full sedative dose of quinia, with the first dose of the bromide, even in the state of pyrexia ; quinia being the antidote to poison. Children seem to tolerate the use of the bromide very well. Give to a child three or four years of age the following : ℞. Potassii bromid. sc. j.; spts. nitri dulc. gtt. v.: aquæ puræ, f oz. ss.; repeat every half hour or hour, according to the frequency of the convulsions. As before said, quinia should be given in sufficient doses to produce cinchonism as early as possible ; the bromide having its great effect in controlling the nervous phenomena. Should the taste be objectionable, add some pleasant syrup.—*Charleston Medical Journal.*

*A Convenient Local Anodyne.*—℞. Elastic colodion ℥j.; hydrochlorate of morphia grs. xv.; dissolve the morphia in the collodion, and spread with a camel's hair brush, some of this solution over the painful part, cover with oil silk. The effect is said to be very satisfactory.—*Lancet.* H. B. L.

*Varicose Vein treated by a new Operation.*—Mr. Marshall, of London, mentions the case of a man who had suffered since he was sixteen years of age with a varicose condition of the left saphenous vein. For the relief of his trouble an operation was performed in November last, and it is described as follows: The patient was first directed to stand for half an hour with a carbolized cloth around the limb; chloroform was then administered, and the course of the tortuous vein was marked in ink. At each end of this mark the vein was ligatured in the following way: a pin was passed under the vein, a piece of bougie over it, and a figure-of-8 suture applied, bringing the two together so as to close the vein; two other similar ligatures were placed above the upper one at a distance of two inches below the lower one. The pins, bougie, and threads had all been previously carbolized.

Esmarch's elastic bandage was then applied to the whole limb, with the effect of completely emptying the varicose vein, even in the intervals between the several pins. A straight incision through the skin, from the top to the bottom of the ink-mark, was then made, and with a knife and director, the vein was slit up along the whole of the exposed portion. As the vein then formed a very large, irregular, folded mass at the bottom of the wound, a ligature was passed around it above and below, and it was then removed with forceps and scissors, two large veins which opened into it being cut during the operation. A strip of carbolized gauze was then placed in the wound, and the limb put up in the complete antiseptic dressing. The operation was done under the carbolized spray. No bleeding whatever occurred. Unfortunately, erysipelas and suppuration, requiring an incision, occurred about one month after the operation; otherwise the patient did well. He was discharged in January last, with the diseased vein satisfactorily obliterated above and below the part that had been removed.—*The Lancet, January 23d*, 1875.

*Worms.*—℞. Santonini, gr. iii.; Sacch. alb., q. s. M. Ft. in chart. no. iii. For a child of two and a half years one of these powders may be given at bedtime for three days, the food in the meantime being soups and broths in small quantity. On the morning of the fourth day a dose of calomel and jalap may be given; subsequently a tonic.

*Two Cases of Idiopathic Tetanus—Treatment by Chloral—Recovery.*—The *Indian Medical Gazette,* February 1, contains the following cases:

The first, a boy 13 years of age, was treated by Sugeon-Major G. T. Hunter for tetanus, occurring after exposure to the sun and beating with a cane. No contusions or wounds could be discovered. Two peculiarities were noticeable: 1. The tetanic spasm commenced at the diaphragm, then affected the fingers of the right hand, and afterwards extended to the muscles of the abdomen and legs. 2. Deglutition as regards liquid nourishment was easy throughout the course of the disease, though he cou'd not open his mouth more than half an inch for several days. Treatment: tr. cannabis indicæ, *m* x. and potass. bromid., grs. v. every third hour, and chloral hydrat. grs. xii. three times a day, together with inhalations of chloroform as required. The diet was generous, and the boy took as much as twenty-two ounces of wine daily, without any intoxicating effect. Recovery took place in about a month.

The second case, under care of Surgeon H. W. Hill, was that of a Hindoo, 40 years of age, who suffered from a severe but typical attack of idiopathic tetanus, caused probably by exposure to the damp chilly air of night, when the body was relaxed by the heat of the day and the constitution rendered "below par" by a recent febrile attack.

During the first few days the tetanic spasms were exceedingly frequent and severe, but these gradually yielded to the steady administration of chloral hydrate, grs. xx. thrice daily, which was kept up through the whole course of the disease, which terminated in recovery at the end of six weeks.—*Med. Times.*

*Nitrite of Amyl.*—Dr. Edwards, in *Viryinia Medical Monthly*, stated that during the past three months he had been using this agent in the case of epilepsy (marked by distinct stomach aura occurring a few minutes before each convulsion) with the effect of preventing the convulsion when taken (2 drops mixed in water) at the commencement of the aura. He mentioned the points made by Dr. Mitchell. Dr. McGuire had used amyl in a case of dysmenorrhœa, but the subject was not a good one, or something else prevented the benefit he had hoped to derive from its use.

*The Couchard.*—Dr. Diver, of Caterham, has invented a very ingenious contrivance for exerting pressure on the abdomen and back of parturient women, which he styles the "couchard." It is a kind of apparatus which the woman puts on during labor, and the patient is enabled, by pulling during the pains, to give considerable pressure to the back and support to the uterus at the same time. The patient puts on a pair of connected pads, one over the back, the other over the front of the body, and then places her feet in a pair of stirrups. Through the tops of these stirrups a cord running from the handle passes so that the hands and feet act in antagonism. This tightens the cord, which is attached to a soft cushioned pad over the back, which draws that pad and the one in front together, and gives great relief to the patient.

Patients have expressed themselves as greatly pleased by the support this very ingeniously devised apparatus affords to them during labor. Dr. E. Diver has taken much trouble in perfecting this invention of his, and writes us that additional experience shows him how much relief it gives to many of his patients. We should mention that, by means of a strong cord attached to the handle, and pulling over the end of the bed, the patient can employ the same abdominal and back pressure comfortably whilst lying in bed. All such devices for assisting in the alleviation of suffering are highly commendable, and we wish success to Dr. Diver's Couchard. —*Medical Press and Circular.*

*The "Tractile Method" in Hernia.*—At a recent meeting of the Société de Chirurgie of Paris, the President, M. Perrin, communicated the particulars of a case of strangulated inguino scrotal hernia which had been reduced by this method. Taxis, under chloroform, had been tried without success by M. Gosselin, and that distinguished surgeon was about to operate, when M. Perrin proposed that it should be tried. Accordingly, forty-six hours after the strangulation had occurred, a hospital attendant took the patient up by his legs, and placing them over his (the attendant's) shoulders, raised him up so that the patient's head and shoulders rested upon the bed. In this position M. Perrin practiced the taxis, and the hernia was soon reduced to half its former size. The patient was replaced upon his bed, and the reduction was completed in the horizontal position.

*Successful Transplantation of Bone.*—The want of success that has attended previous attempts at transplanting portions of bone renders this case of Prof. Nussbaum's particularly interesting. An officer, in 1870, received a gunshot wound which resulted in a false joint in the middle of the right ulna. The fractured ends were connected by a thin cicatrical cord. The functions of the limb were seriously impaired, notwithstanding the integrity of the radius. To relieve the deformity, the following operation was done, the age of the patient at the time being twenty-four: The false joint was laid bare, and the cartilaginous ends of the bones, which were pointed, together with the false ligament, were removed by strong scissors. The upper part of the ulna was then sawed half across about two inches above the end of the bone, and the upper piece, together with its periosteum, split off with a hammer and chisel. This was so managed, however, as to retain a small bridge of the periosteum for the nutrition of the bone. Finally, the detached piece was so placed in the vacant space that its upper surface was now external, its lower internal, and its outer superior. The fatty and indurated soft parts were divided so as to set up an inflammatory reaction, the bleeding checked by a stream of carbolized water, the wound closed by sutures, and a fenestrated plaster-of-Paris bandage applied.—One small spiculum of necrosed bone subsequently came away. The patient made a perfect recovery, so that in the following year he had such full use of his arm that he was appointed to a regiment.—*Aerztl. Int. Bl., Feb.* 23, 1874.—*All. Wien. Med. Ztg.*

*Remedy for Cholera.*—During the late cholera epidemic in Vienna, a new remedy called camphorein, was used with great success in the hospitals. It is prepared simply by passing chlorine gas into pure turpentine oil until saturated; it gives a thick, heavy, oily fluid, of brown color, with a strong smell of chlorine. This is freed from muriatic acid by washing with water. The remedy is applied by placing a portion in a flat vessel and holding it to the patient to inhale.—*Eclectic.*—*Pen & Plow.* H. B. L.

*Carminative Mixture for Young Infants.*—℞. Sodii bicarbonat., gr. iss.; spirit. ammon. aromat., gtt. ii.; glycerinæ, gtt. v.; aq. menth. pip., f℥ss. M.

*Inunction as a Remedy in Measles and Scarlatina.*—J. L. MILTON, Senior Surgeon to St. John's Hospital, London.—Dr. Milton begins his paper by regretting his failure to introduce this treatment into England. He gives twelve cases of measles, most of them complicated, and running a severe course, treated by this method. One was dying when he first saw her, the rest recovered. He directs a piece of fat bacon, well washed, to be exposed to heat, and the patient rubbed with this from head to feet, as soon as the fat melts. Repeat the rubbing morning and night, and don't wash the patient at all. The only other treatment used was am. carb. in large doses, chlorate pot., and aperients, given only to meet indications as they appeared. He gave brandy and beef-tea freely in the worst cases. The same treatment for scarlatina. The advantages claimed are, more speedy recovery, and almost perfect prevention of infection to persons about the room. He is afraid to risk this for the room and bed but directs these to be completely disinfected. —*Archives of Dermatology, April* 1875. H. B. L.

*Transparent Turpentine Liniment.*.—The following preparation is so composed that, instead of making the half-dissolved cloudy mixture usually seen where oil of turpentine has been mixed with tinctures, it forms a perfectly clear solution : ℞. Ol. terebinth., tinct. aconit. rad., spiritus camphoræ, aa ℥ii.; Tinct. iodinii., carbon bisulph., aa ℥i. M. This mixture separates very quickly into two layers ; but if the following substances are added it becomes perfectly clear: Soap, transparent glycerin, in fine shavings, ¾ oz ; otto of cedar, 1½ oz.; or otto of cajeput, 1 oz. Either of these may be used, and if the bisulphide of carbon is deodorized, the liniment is not offensive to the smell.

*Prolapsus Ani treated by Ergotin.*—Von Langenbeck is said to treat prolapsus ani with astonishing success by hypodermic injections of a solution of ergotin (five to fifteen parts in one hundred of water). He replaces the bowel, and, inserting the point of the syringe about an inch deep into the cellular tissue, throws in from one to two grains of ergotin. This should be repeated every three or four days for several weeks, hardened fecal masses having been first removed by injection.—*Medical Press.*

*Wounds Dressed with Silicated Cotton.*—M. Ollier has addressed a note to the French Academie des Sciences, and states that he finds great advantage in the use of the cotton dressing. One advantage is, that the dressings are made less frequently, which is important, because after each such occasion the temperature of the patient rises, and besides, the wound is exposed to the air, and consequently to the infectious germs that are in the atmosphere. But there is another advantage, which is, that, considering the matter of the absorption of infectious germs, it is important not to break the granulous membrane that covers the wound, while frequent designs are apt to do this. To obviate such a danger more fully, he advises that the cotton be covered with a silicated bandage, so that while the dressing occludes the germs completely, as it appears to, it also is immovable. He urges that this improved method may prove to be very useful in army practice, in the transportation of the wounded. —*Jour. de Méd. et de Chir., March,* 1875.

*Explosive Medicines.*—Doctors are warned (*Revue de Therap.,* 1873) against combining certain agents, which in case of development of acid, or if exposed to moderately high temperature, are liable to *go off.* Among these dangerous formulæ may be cited a prescription for pills not unfrequently employed in England, composed of nitrate of silver, extract of nux vomica, muriate of morphia, conserve of roses and extract of gentian, which, when affected by the development of heat, will speedily explode. In like manner, pills made of nitrate of silver and creosote, or carbolic acid, will very soon generate heat, sufficient to induce spontaneous combustion. Still more surprising to the occupants of the sick chamber is the energetic explosion (suggestive of nitro-glycerin) arising from the pills or mixtures of which oximuriate of potash forms an ingredient.—*Drug. Price Current.*

*Dysmenorrhœa.*—Dr. Horner, in *Va. Med. Monthly,* strongly recommends the following prescription to relieve the pain of acute dysmenorrhœa: ℞. Ext. belladonnæ, gr. xij.; ext. conii., ℈ij.; morphiæ sulphat., gr. vj.; plumbi acetat., gr. xij.; butyr. cocoæ., ʒvj. M. Make suppositories No. xii. S. One to be used at bedtime—the bowels to be evacuated the following morning by the use of Davidson's syringe.

*Belladonna in the Treatment of Profuse Perspiration.*—Anthony Butler, M. B., Assistant Medical Officer, Town's Hospital, Glasgow.—In corroboration of the estimate of the value of belladonna in checking excessive perspiration in different diseases, pointed out by Dr. Ringe in his Therapeutics, I beg to record the results of my experience with its alkaloid atropia in this condition. Since July last, in the wards of this institution to which I am especially attached, I have prescribed it to upwards of thirty patients, most of them suffering from phthisis pulmonalis, but a few from other diseases. It was given at bedtime in pill, in doses of one-eightieth of a grain. The results have been very encouraging. In about one-half of the whole number of patients, after from one to four pills, the perspiration was either checked altogether or diminished in amount. In other cases no decided effect was produced till it had been used for about a week or ten days. In about a third of the cases no apparent benefit resulted, and the medicine was discontinued. In some of these cases the perspiration did not recur in a few instances, even after the medicine was stopped; but in others it returned, and was again checked when the pills were resumed.—The patients themselves were so convinced of its beneficial influence that they often asked for their pills when they had been omitted for a time. Caution, however, requires to be used in administering this drug, as in two of my patients distinct toxic effects were produced by the doses mentioned.—*British Medical Journal.—Physician and Pharmacist.*

*Treatment of Whooping-cough.*—Wild claims that he can cure every case of whooping-cough within eight days by the following treatment: The patient is not to leave the room, and at every access of coughing is to hold before his mouth a small piece of cloth folded several times, and wet with a teaspoonful of the following solution: Ether, 60 parts; chloroform, 30 parts; turpentine, 1 part.—*Deutsches Archiv. f. Klin. Med. Allg. Wein. Med. Ztg.*

*Salicylic Acid for Ulcers.*—This article is now used as a dressing for ulcers, wounds, etc., in the proportion of ʒi. to ℥lxijss. of water. So far, its substitution for carbolic acid has been agreeable and quite satisfactory.—*N. Y. Med. Record.*

*Milk as a Vehicle of Disease.*—Dr. E. W. Gray in a very interesting article on milk published in the *Sanitarian* for May, after proving that the good people of New York "pay annually $2,190,000 to the dealers for diluting and spoiling their milk supply," comes to the following conclusions :

1st. Milk is capable of receiving and propagating at least some of the contagions ; and that it may become infected by the admixture of even a small quantity of infected water, or by absorbing the poison from an infected atmostphere. As the substance of these poisons is not recognizable by the senses, and has not been revealed by the microscope, or by chemical analysis, there exists no means of knowing whether milk is infected and poisonous or not, and hence the necessity of the greatest precaution to prevent exposure.

2d. The milk of diseased cows, or such as are not in good physiological condition, from irregular and bad feeding, from abuse, overheating and excitement, or from any cause whatever, is always unsafe and dangerous; and hence the necessity of generous feeding with good wholesome food, and habitual leisurely handling and tender treatment, in order to obtain a good and perfectly safe article of milk.

3d. That milk will absorb from the atmostphere, offensive and hurtful odors and poisonous gases, if such exist in contiguity, and hence the necesssity of keeping it in good cool well-ventilated apartments, well removed and protected from all odoriferous and decaying matter, and in vessels kept scrupulously clean and sweet by frequent ablutions in boiling water.—*Sanitarian.*

*Chronic Dysentery.*—Cases of chronic dysentery, such as obtain in the course of phthisis or the ordinary cases of the disease, have been treated of late by administering—℞. Ol. ricini, ℥ss.; tr. opii, *m* xx. M. Three times a day. The success attending this method of treatment had been very satisfactory.—*N. Y. Med. Record.*

*Infant Food.*—Dr. Hamlin, of Maine, says, that "healthy and selected cow's milk, with the addition of a little cooked and strained oat-meal and a spoonful of lime water, is a safer diet for infantile life than the milk of one-half the mothers of the present day." He does not dilute or warm the cow's milk.

*Bromide of Potassium as a Caustic, Anæsthetic and Curative Application to Malignant, Suppurating Tumors.*—Peyrand (*Il Raccoglitore Med.*) had a cancroid of the face which produced excruciating pains, and which could not be extirpated. He determined to sprinkle the surface with finely pulverized bromide of potassium, after doing which the pains diminished in intensity, and the growth of the fungous granulations was checked. This treatment was continued for four weeks with excellent results, and during this time the tumor entirely subsided. In this case the bromide exerted an anæsthetic influence on the terminal fibres of the sensitive nerves, diminishing the pain, and acted also through its vaso-motor peculiarity, by contracting the vessels or the tumor and thus diminishing its volume. It acted finally as a caustic, and destroyed the superficial tissue; upon this point Peyrand advises that in order to obtain the full effects of the bromide, the surface should be gently washed off just previous to the sprinkling of the powers.—*Allg. Wien. Med. Zeitung.*

*A Hint in Giving Iodide of Potassium.*—A useful hint is revived in the *British Med. Journal* by Mr. Joseph P. McSweeney. He says: "Sir James Paget was the first to call the attention of the medical profession to the following interesting fact—namely, that carbonate of ammonia greatly increases the therapeutic action of iodide of potassium. I have had extensive experience in the treatment of syphilis, and have tried it with the best results, and find that five grains of potassium, combined with three grains of carbonate of ammonia, are equal to eight grains of the potassium salt administered in the ordinary way."—*Medical Examiner.*

*Too Good to be True.*—M. Paraf has discovered a way of doing without rain, if necessary. He knew that the air is full of moisture, and he knew that chloride of calcium would attract and condense it for cultural purposes. He has applied this chloride on sand-hills and road-beds, on grass, on all sorts of soils, successfully, and he has ascertained that it may be applied in such proportions as will produce the irrigation of land more cheaply and efficiently than by means of canals or other methods of securing artificial irrigation.—*Boston Journal of Chemistry.*

*Copaiba in Dropsy.*—Dr. Falconer (*Canada Lancet, Dec.* 1874) feels confident that very few practitioners have given this drug a fair trial in ascites. Passing by cases of cirrhosis and chronic peritonitis, with their accompanying effusions, in which, after reaccumulation after tapping, he has succeeded in not only diminishing but in completely removing the fluid by the use of copaiba, he refers to a case of ovarian dropsy, which he treated successfully by the drug. Copaiba is the diuretic, par excellence, and never fails in removing the serum through the kidneys, except in chornic albuminuria. It obviates the use of elaterium or gamboge, tapping or ovariotomy, in many cases.

*Treatment of Galactorrhœa.*—Dr. Vacher (*France Medicale*) has recently made a report on this subject, and has given the details of several cases in which he found the best curative results to follow the continued administration of iodide of potassium. The doses should at first be small, but are to be gradually increased until the desired effect be obtained. He found that the flow did not cease until symptoms of mammary congestion made their appearance. —*Allg. Wiener Med. Zeitung.*

*Administration of Castor Oil.*—M. Potain recommends, as the best method of concealing the unpleasant flavor of castor-oil, to squeeze half an orange into a glass, and pour the oil upon it; then, avoiding all disturbance of the liquids, to squeeze the juice from the other half of the orange carefully over it. The oil, thus enclosed between two layers of orange-juice, can be swallowed without the least perception of its flavor.—*Medical Times.*

*Formulæ for the Troublesome Cough of Phthisis.*—℞. Potassii bromidi, potassæ chloratis, ammoniæ muriatis, aa ʒ iss.; syrup. tolutani, ℥ iv. M. Tablespoonful every two or three hours.

℞. Tinct. opii camph., ℥ i.; tinct. belladonnæ, ʒ i.; tinct. hyoscyami, ʒ ij.; spiritus lavendulæ comp., ℥ i. M. Ten drops on a lump of loaf sugar every hour until cough is relieved.—*Charity Hospital, New York.*

*Pills of Iodide of Potassium.*—℞. Potassii iodid., ʒiss.; pulv. althæ, ʒiss.; syrupi, q. s. M. Ft. pil. No. xii.—*Med. Times.*

*Arrest of Erysipelas by Means of Injections of Carbolic Acid.*—A case of the above was one of gunshot wound of the knee. Erysipelas entered it and began to spread up and down the limb. The limb was girdled with hypodermic injections about one inch beyond the border of the erysipelatous blush. The strength of the solution used was 2 per cent. The first time the erysipelas marched over the barrier. The second line of injections arrested it, or rather the erysipelas did not spread beyond them. The house surgeon remarked that injections of this character had evidently been of service to many cases of the same kind in his ward.—*Med. Rec.*

*Ergot in Acute Mania.*—Guided by the observations of Brown-Sèquard, that contraction of the meningeal vessels and anæmia of the brain followed the administration of ergot, Dr. van Andel (*Schmidt's Jahrbucher*, Feb., '75.,) has employed it in several cases of acute mania, and with the most satisfactory results. He relates several cases both of this disease and of other hyperæmic states of the nervous centres in which it caused very rapid amelioration of the threatening symptoms. He prefers the hypodermic method, and employs a solution of ergotin in alcohol and glycerin.—*Clinic.*

*Trichaniæ in Wild Boars.*—A boar between six and eight years old was lately shot in the forest of Saxony, and when its muscular tissue was microscopically examined it was found to be thickly studded with trichinæ. This case is of interest as being the first in which parasite has been found in the wild boar, it having been the general belief that the domesticated animal was the only one affected.—*Allg. Wein. Med. Zeitung.*

*Liniment for Scabies.*—Dr. Clements gives the following formula: ℞. Acid. arsenios., gr. i.; potassii carb., gr. xv.; spiritus saponis, f℥iii.; aquæ, f℥iii. M. The liniment to be rubbed twice daily on the part affected. It does not harm the youngest child.—*Medical Times.*

*Anti-Gastralgic Pills.*—℞. Ext. belladonnæ, gr. vi.; quiniæ sulph., ʒi. M. Ft. in pil. No. xxv. Sig. Thrice daily, in the treatment of gastralgia.—*Medical Times.*

*Injection in Gonorrhœa.*—Dr. J. Bligh, in *La Union Med.*, proposes the following: ℞. Potass. bromid., ℨ iss.; glycerinæ, f℥ iij.; aquæ destillat., f℥ v. M. S. Inject every four hours. Bromide of potassium is, at the same time, given internally. The author prescribes this injection, not only in chronic gonorrhœa, or in the sub-acute stage, but also in the acute stage, and especially in chordee. In the final stage, an astringent may be added to this solution if thought proper. Abstinence from beer and all stimulating drinks is enjoined; emollient ptisans, if the urine is not abundant.—*Le Progres Medical.*

*Treatment of Persistent Neuralgia.*—Among the many remedies that have been tried for rebellious neuralgias, M. Desnos, of the Hôpital de la Pitié, recommends the following combination as being frequently successful; and even in cases where it has failed, if tried again after the lapse of a short time, it may succeed. He first applies over the painful spot three or four mustard poultices, and then rubs into the reddened surface a liniment composed of—℞. Oil of hyoscyamus, ℥ iss.; laudanum, ℨ ss.; chloroform, ℨ iss. M.—*Journal de Medicine.*

*Bromide of Calcium in the Nocturnal Pains of Syphilis.*—Dr. Galozzi and Prof. Gamberini have employed the bromide of calcium as a sedative in the nocturnal pains of syphilis, with considerable success. The formula employed by them is the following: ℞. Calcis bromid, gr. iv. ad. ix.; aquæ destillat., ℥iv.; sacch. alb., ℨii. The dose to be increased by 1½ gr. daily. —*Med. Times.*

*New Antiseptic Ointment.*—Professor Lister, according to the *Student's Journal and Hospital Gazette*, is at present using, with great success, an ointment composed of paraffin, 2 parts; white wax, 1 part; sweet oil of almonds, 2 parts; and boracic acid (powdered), 1 part.—*Med. and Surg. Reporter.*

*Pills of Aconite.*—℞. Ext. aconiti, gr. vii.; confect. rosæ, q. s. M. Ft. in pil. No. xx. One to two, morning and evening, in the osteocopic pains of syphilis, combined with the usual anti-syphilitic treatment.—*Med. Times.*

*A Pill for Jaundice.*—Dr. Bartlett writes to the *Missouri Clinical Record*: The following combination I use in icterus, or jaundice: ℞. Hydr. chlor. mit., gr. iij.; quiniæ sulph., gr. xxiv.; Pulv. opii., gr. jss. M. Ft. pil. No. 12. Sig. One pill every four hours. This pill will act when a dose of calomel unencumbered fails to act.—The opium, of course, confines it, but the quinia also materially assists its action.—*Medical & Surgical Reporter.*

*Treatment of Pertussis by Inhalation.*—℞. Ext. belladonnæ *m* v. ad x.; potass. bromid., ℈i.; ammon. bromid., ℈ii.; aqua, f℥ii. M.—Inhale one tablespoonful in the ordinary steam atomizer; or this amount may be diluted by filling up the glass with water. In severe cases this may be used twice daily, until the urgency of the symptoms is relieved, and then continue once daily until the cough has entirely disappeared.—*Boston Med. and Surg. Journal.*

*Unchangeable Solution of Quinia for Hypodermic Injections.*—℞. Sulphate of quinia, 15 grains; Lactic acid (concentrated), 15 grains; water, sufficcient to make 60 minims; Bisulphite of sodium, ½ grain. When the solution of the quinia is perfect, add the bisulphite of sodium, and transfer to a well-corked phial—*A. P. Sharp, in Proc. Am. Pharm Association*, 1874.

*Prescription of Cures for Pharyngeal Diphtheria.*—℞. Pulv cubeb. (fresh), ʒx.; glycerinæ, ʒxviii.; mel. despumat., ʒviiss.; aq. menth., ℥iii. ʒi.; gum. tragacanth., gr. xv.; tinct. ol. menth. pip., gtt. viii. M. Dose for a child, one teaspoonful every hour; the broken doses producing the desired effect without irritating the digestive tract.—*La Tribune Medicale.*

*Liquid Glycerin for Burns.*—℞. Calcis oxid., gr. iii.; spirit. chloroformi, gr. iii.; Glycerinæ, fʒiiss. M. Charpie is dipped in the mixture, and placed over the burned surface; it is then covered with a thin sheet of gutta-percha, and the whole surmounted with a loose bandage. It is important that the charpie should be closely applied to the entire burned surface. The pain ceases almost instantly, and the sore heals very rapidly.—*Trans. N. Y. Medical Journal.*

*Ether for Tape-Worms.*—When the anesthetic power of either was first discovered, it was only proposed to use it on human beings to render surgical operations painless. Von Heyden, the merciful man who would not inflict pain on any living creature, employed it as long ago as 1830 for killing insects for his collection. Even worms are rendered dormant and helpless by its use. Prof August Vegel now announces a new application of this anesthesia for worms—its application to tape-worms. The ether is vaporized in the stomach, and the worm stupefied, it being then easily removed by any of the usual remedies, against which, when awake, the worm offers a strong resistance.—*Med. Examiner.*

*External Use of Turpentine in the Treatment of Tonsilitis.*—In the *Leavenworth Medical Herald* Dr. S. H. Roberts strongly recommends the use of turpentine externally in tonsilitis. He folds the flannel to four thicknesses, wrings it out in hot water, and pours oil turpentine over a spot the size of a silver dollar. The flannel is then applied over the sub-parotid region, and the fomentation continued as long as it can be borne. After removal a dry flannel is applied, and the same region rubbed with turpentine every two hours. This application is continued daily till resolution occurs. The doctor believes, from the evidence of his long experience, that thus applied early in the disease, the oil of turpentine has almost a specific effect in tonsilitis. That its action is not simply that of an irritant, he has proved by employing mustard, croton oil, tr. iodine, etc., in the same class of cases. They always failed to diminish the inflammation of tonsils, while the turpentine succeeded.—*Detroit Review of Medicine.*

*Formula for Hay Fever.*—℞. Potassæ, gr. xx.; morphia sulphatis, gr. iv.; aquæ destillatæ, ℥ij. M. This mixture, to be used by means of the atomizer, is recommended by Dr. Hoover as giving immediate relief, and producing a complete cure within a few days. —*American Journal of the Medical Sciences.*

*Method of Administering Raw Meat.*—Raw meat, scraped, 3 drachms; pulverized sugar, 12, drachms; claret wine, 6 fluid drachms; tincture of canella, ½ drachm.—*Medical Times.*

